# Design of Polyether sulfone flat-sheet membranes for multi-layered hemodiafiltration devices

**DOI:** 10.1371/journal.pone.0324498

**Published:** 2025-06-20

**Authors:** Rei Kono, Takashi Ota, Yoshihiko Kanno, Norihisa Miki

**Affiliations:** 1 Department of Mechanical Engineering, Keio University, Yokohama, Kanagawa, Japan; 2 Department of Nephrology, Tokyo Medical University, Shinjuku-ku, Tokyo, Japan; NED University of Engineering and Technology, PAKISTAN

## Abstract

Multilayered hemodialysis devices enable blood flow without the need for an external pump, whereas hollow fiber-type devices require a pump due to significant pressure loss across the fibers. This highlights a key advantage of multilayered devices for implantable applications, where a simpler and lighter system reduces the burden on patients. This study investigates the mechanical strength and dialysis efficiency of polyether sulfone (PES) flat-sheet membranes for multilayered devices. PES membranes, with thicknesses ranging from 40 to 160 µm, were prepared using the liquid inversion method, with thickness controlled via spin coating. The mechanical strength of the membranes was tested following the ISO 8637–1:2017 protocol, and membranes thicker than 80 µm were experimentally verified to withstand pressures of up to 500 mmHg, making them suitable for dialysis applications. Furthermore, the study demonstrates the successful use of 80 µm membranes in both in vitro and ex vivo experiments with rats, identifying this thickness as optimal for multilayered dialysis devices.

## Introduction

Hemodialysis treatment is the most conventionally used renal replacement therapy [1]. Dialysis membranes, which allows the wastes and water to be removed from the blood while maintaining proteins and other large molecules, determines the dialysis performance [2–4]. A wide variety of membranes are commercialized, which include polyether sulfone, polyacrylonitrile, cellulose triacetate and polyvinylpyrrolidone: polyarylethersulfone, and ethylene vinyl alcohol copolymer [5–9].

Most of the commercially available dialyzers have hollow-fiber membranes due to the large surface-to-volume ratio, ease of manufacturing, and robustness against blood leak. The dialysis console pressurizes the blood with a pump up to 150 mmHg (20.0 kPa) and let the blood flow through the fibers, which are typically 200 μm in diameter and 16–30 cm in length. The thickness of the typical hollow-fiber membrane ranges from 10 to 50 μm [2].

There is a strong demand for implantable and wearable dialysis devices, as they reduce the frequency of hospital visits and significantly improve patients’ quality of life. Such applications require a minimally complex system to enhance robustness and reduce the burden on patients. Hollow fiber-type devices necessitate an external pump to introduce blood due to the significant pressure loss across their narrow fibers. Such pumps require electricity, add complexity and weight to the system, and should be avoided. Additionally, hollow fibers may impose high mechanical stress on the blood as it passes through tens of thousands of fibers before merging into a single flow, increasing the likelihood of blood coagulation. Since implantable devices are intended for long-term use compared to conventional hemodialysis devices, minimizing blood coagulation is crucial for their effectiveness and safety.

Multi-layered hemodialysis devices, which are composed of micro-channels for blood and filtrates or dialysis fluids and flat-sheet dialysis membranes, can operate at low pressure due to the small pressure loss. Our group has been studying the multi-layered hemodialysis/filtration devices that can work under blood pressure without any external pumps for the implantable applications in mind [10–13]. Polyether sulfone (PES) is used for the membranes considering its high biocompatibility and low adhesiveness to biomaterials. The amount of diafiltration can be adjusted by the number of the layers.

As discussed later, the flat-sheet membranes involve larger stress than the hollow-fiber membranes when the applied pressure and the membrane thickness is the same, i.e., the higher mechanical strength of the membrane is required for the flat-sheet membrane. The formed PES membrane is known to have an asymmetric structure, consisting of a nano porous skin layer and a support layer with microstructures [10,14–17]. Since the skin layer has nano-sized pores, the filtration capacity of the membrane depends on the thickness of the skin layer, whereas the thickness of the membrane determines its mechanical strength. A PES membrane is fabricated by the liquid inversion method in our study, where the thickness of the membrane can be controlled by spin-coating process of the PES casting solution.

In this paper, first, we discuss the differences between the flat-sheet membranes and hollow-fiber membranes used in the dialysis/filtration applications with respect to the mechanical properties. Then, we experimentally investigate the PES membranes with different thickness through pressure tests and *in vitro* and *ex vivo* hemodiafiltration experiments. The optimal membrane thickness will be deduced to design the implantable hemodiafiltration devices.

## Materials and methods

### Device design

**Fig 1:**
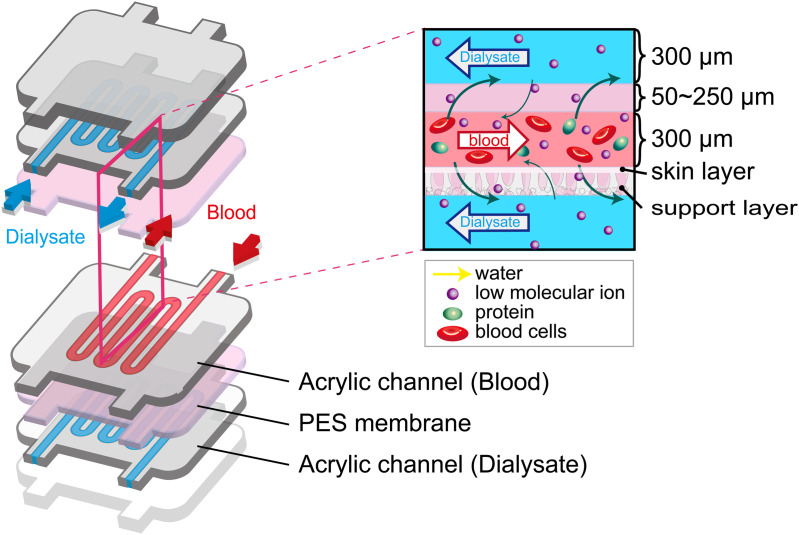
Illustration of the multi-layered hemodiafiltration device.

Fig 1 illustrates the multi-layered hemodiafiltration device, in which small molecular-sized waste products are removed from the blood to the dialysate through the membranes. The microchannels 300 µm in height for the blood and the dialysate are separated with the PES membranes.

### Material and device fabrication

**Fig 2:**
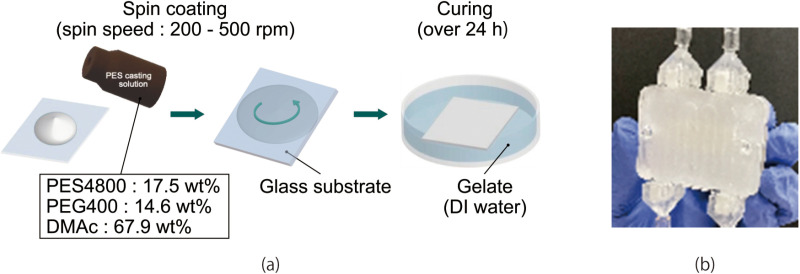
(a) Fabrication process of PES membrane. (b) Photograph of the fabricated device.

The fabrication processes are shown in Fig 2. PES membranes are formed by the liquid inversion method [14]. First, PES with a molecular weight of 4800 (Sumitomo Chemical Co., Tokyo, Japan), polyethylene glycol with a molecular weight of 400 (Fujifilm Wako Chemical Corporation, Osaka, Japan), and dimethylacetamide (Fujifilm Wako Chemical Corporation, Osaka, Japan) were mixed in proportions of 17.5wt%, 14.6wt% and 67.9wt%, respectively, at 60 °C. The solution was then cooled to room temperature and stored. The membranes formed with PEG were experimentally verified to possess sufficient water permeability, which is essential for hemofiltration applications [12]. 250 mL of the PES casting solution was sufficient for forming 10 membranes.

Next, the PES casting solution was spin-coated onto a square glass substrate (100 mm × 100 mm), with the spinning speed determining the resulting membrane thickness. The volume of the solution was not precisely controlled during the spin-coating process. In our experiment, membrane thicknesses ranging from 40 to 250 µm were tested, corresponding to spinning speeds from 500 to 200 rpm. The spinning speed was maintained for 30 s. Within 5 s after the spin-coating process was completed, the substrate was immersed in deionized (DI) water for 24 h, allowing the PES membranes to form. The thickness of the membrane was measured using a digital micrometer.

For ESEM measurements, PES membranes with thicknesses of 50, 100, 150, 200, and 250 µm were evaluated, while 40, 80, 120, and 160 µm thick membranes were examined in the pressure resistance tests. Next, the substrate was carefully immersed into the deionized (DI) water for 24 h while the PES membranes were formed.

Two microfluidic channels for the blood and the dialysate are formed from 0.3 mm acrylic plates using a laser cutting machine (Speedy 300, Trotec Laser Japan, Tokyo, Japan).

Finally, the PES membrane, dialysate flow channel, and blood flow channel are assembled to form seven-layers device using cyanoacrylate adhesive (LOCTITE 4304/4305, Henkel Japan, Tokyo, Japan). The skin layer of the PES membrane faces the blood channel, while the support layer is in contact with the dialysate/filtrate channel. The assembled device is optically inspected to ensure that the adhesive is correctly applied.

In the following experiments, several solutions are used; Bovine defibrinated blood (R100-0050) was purchased from Rockland Immunochemicals, Inc., PA, USA. Saline solution was purchased from Otsuka Pharmaceutical, Tokyo, Japan. Albumin and creatinine, which were mixed with the saline solution were purchased from Fujifilm Wako Pure Chemical Corporation, Osaka, Japan.

### Mechanical analysis

**Fig 3:**
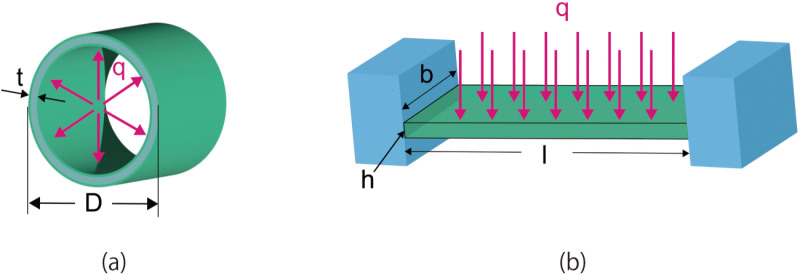
The 3D models of (a) hollow fiber membrane and (b) flat-sheet-sheet membrane.

The mechanical stress applied to the conventional hollow fiber membranes and the flat-sheet membranes in the applications of dialyzers are discussed here. They are regarded as thin-walled cylinders and beams fixed at both ends, as shown in Fig 3.

The circumferential stress $\sigma_{\theta }$ for the hollow fiber membranes and the maximum stress exerted on the beam $\sigma_{\max}$ can be expressed as,


$$\sigma_{\theta }=\frac{PD}{2t}\;$$ (1)

$$\sigma_{\max}=\frac{M_{\max}}{Z}=\frac{ql^{2}}{2bh^{2}}$$ (2)

where P [MPa] is the internal pressure, D [mm] is the inside diameter, t [mm] is the wall thickness, $M_{\max}$ [N⋅mm] is the maximum bending moment, *Z* is the section modulus [mm^3^], q [N/mm] is the equally distributed load, l [mm] is the width, b [mm] is the length, and h [mm] is the height. $\sigma_{\max}$ increases with the width and decreases with the thickness. The maximum stress is exerted at the edges of the channel.

When the applied pressure is the same, the ratio $\frac{\sigma_{\max}}{\sigma_{\theta }}$ is expressed as,


$$\frac{\sigma_{\max}}{\sigma_{\theta }}=\frac{l^{2}t}{h^{2}D}\;$$ (3)

The conventional hollow fiber membranes have a diameter D of 0.2 mm, a membrane thickness t of 0.04 mm [2]. In our device design, the width of the channel l is set to be 2 mm. When the ratio $\frac{\sigma_{\max}}{\sigma_{\theta }}$ is 1, the thickness of the flat-sheet membrane h is 0.89 mm, which is significantly larger than the thickness of the hollow fiber. As will be described below, this increased thickness may result in a smaller diffusion capacity. Therefore, the thickness needs to be determined such that the membrane can withstand the maximum internal pressure, which will be determined in the following section.

### ESEM analysis

PES membranes consist of a skin layer with nanometer-sized pores and a support layer with micrometer-sized pores, which contribute to dialysis performance and mechanical strength, respectively. To investigate the relationship between the total membrane thickness and the thicknesses of the skin and support layers, we optically examined the PES membranes using an environmental scanning electron microscope (ESEM) (Inspect S50, FEI, USA). PES membranes with thicknesses of 50, 100, 150, 200, and 250 µm were analyzed.

For SEM imaging, the membranes were cut into 10 × 10 mm sections using a scalpel to expose the cross-section. The specimens were then attached to the stage with a conductive adhesive and dried for 1 hour. Subsequently, an osmium coating (5 nm thick) was applied before SEM measurement.

### Pressure resistance tests

**Fig 4:**
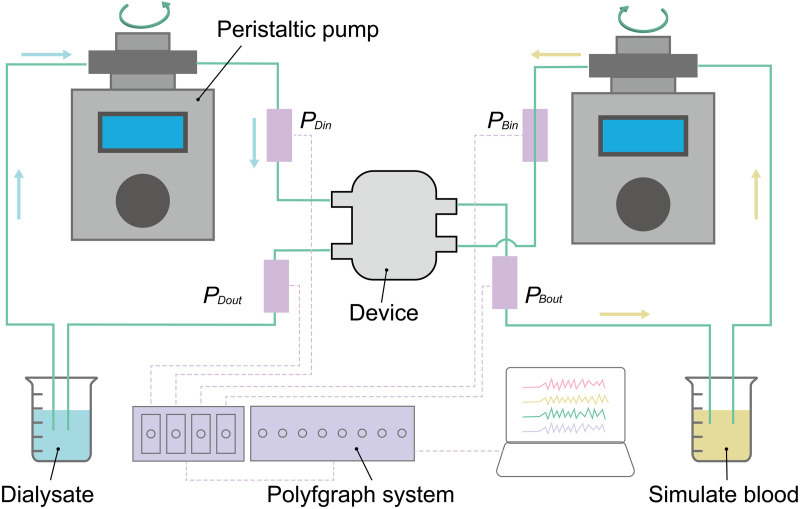
Illustration of the experimental setup for pressure resistance test and dialysis performance test. Pressures at the inlet and outlet of the device are ($P_{Bin}$ and $P_{Bout}$ for the blood channel and $P_{Din}$ and $P_{Dout}$ for the dialysate channel) and measured with pressure gages and a polygraph system.

Pressure resistance tests were conducted to assess the mechanical strength of the PES flat-sheet membranes, following the procedure outlined in ISO 8637–1:2017, Extracorporeal Systems for Blood Purification – Part 1: Haemodialysers, Haemodiafilters, Haemofilters, and Haemoconcentrators. Triple-layered devices with one blood microchannel and two dialysate microchannels were fabricated using the PES flat-sheet membranes with thicknesses of 40, 80, 120, and 160 µm. The experimental circuit shown in Fig 4 was formed using a peristaltic pump (PeriStar Pro; World Precision Instruments, Sarasota, FL, USA), pressure gauges (DX-100; Nihon Kohden, Tokyo, Japan), a blood pressure monitor (RMT-1000MG; Nihon Kohden, Tokyo, Japan), extension tubes (SF-ET2022L; Terumo, Tokyo, Japan) and the device. Bovine defibrinated blood (Rockland Immunochemicals, Inc., PA, USA) and saline solution (Otsuka Pharmaceutical, Tokyo, Japan) filled the blood and the dialysate channels, respectively. The pressure inside the device was gradually increased while the outlet of the blood microchannel was cramped. When the rupture takes place, the blood in red color is observed in the filtrate channel. The pressure at which the PES flat-sheet membrane ruptured was recorded, where the maximum detection limit of the system was 88 kPa (660 mmHg). In general, dialyzers are designed to be used with pressures of less than 67 kPa (500 mmHg). The membranes are considered to be acceptable when they do not rupture under 67 kPa (500 mmHg).

The highest reported blood pressure is 360 mmHg [18], while pressures above 222 mmHg are considered critical [19]. ISO 8637–1:2017 recommends applying 1.5 times the manufacturer’s specified pressure. Therefore, we set the threshold at 500 mmHg (67 kPa).

### *in vitro* experiments to evaluate dialysis performance

Before testing the device in animals, as much information as possible should be gathered through in vitro experiments. The dialysis performance of the device is evaluated using the circuit, which is shown in Fig 4, based on ISO 8637. Saline solution with albumin and creatinine as the simulated blood and saline solution as the simulated dialysate are used in the experiments. The initial concentration of the albumin and creatine was set to be 5 g/dL and 11 mg/dL.

Clearance is one of the commonly used indices of the dialysis equipment, which is the removal efficiency of blood etiologic substances, and the creatinine clearance $K_{t}$ [mL/min] is expressed by the following equation,


$$K_{t}=\frac{\left(C_{B(t)}-C_{B(t-1)}\right)}{C_{B(0)}}\times Q_{B}$$ (4)

where $C_{B(t)}$ is the creatinine concentration of the blood at the time t [min] and *Q*_*B*_ [mL/min] is the amount of blood flow.

We also measured the filtration coefficient $L_{p}$ [mL/h mmHg m^2^] to assess the membrane property, which is determined as,


$$L_{p}=\frac{V_{F}}{T_{F}\times TMP\times A}$$ (5)

where $V_{F}$ [mL] is the volumetric amount of filtrate, $T_{F}$ [h] is the filtration time, and TMP [mmHg] is the transmembrane pressure. A [m^2^] is the membrane area. TMP is expressed as,


$$TMP=\;P_{B}-P_{F}-P_{c}=\;\frac{P_{B_{in}}+P_{B_{out}}}{2}-\frac{P_{D_{in}}+P_{D_{out}}}{2}-P_{c}$$ (6)

Where $P_{B_{in}}$ and $P_{B_{out}}$ are the pressures of the inlet and outlet of blood microchannels, $P_{D_{in}}$ and $P_{D_{out}}$ are those of dialysate microchannels, $P_{c}$ is the oncotic pressure caused of albumin and other large molecules in the blood [20–24]. $P_{c}$ is expressed with the concentration of total protein $C_{TP}$ [g/dL] as,


$$P_{c}=\;2.1\;C_{TP}+0.16\;{C_{TP}}^{2}+0.009\;{C_{TP}}^{3}$$ (7)

We tested the PES membranes 80, 120, and 160 µm in thickness, which were found to resist 67 kPa (500 mmHg) as described below. Saline solution mixed with albumin and creatinine (Fujifilm Wako Pure Chemical Corporation, Osaka, Japan) and saline solution were used as the simulated blood and the dialysate, respectively. The simulated blood samples were collected from these circuits every hour for 6 hours and the concentrations of albumin and creatinine were measured using a dry clinical chemistry analyzer (SpotChem EZ SP-4430; Arkray, Kyoto, Japan). The analyzer’s performance is reported to have a coefficient of variation (CV) of less than 4.5% for creatinine and less than 3.0% for albumin, as stated in the FDA 510(k) substantial equivalence determination (510(k) number: K053401). The creatine clearance $K_{t}$ was calculated by Eq. (4) and the filtration coefficient $L_{p}$ was calculated by Eq. (5).

### Creatinine removal rate for *ex vivo* experiments

In the case of *ex vivo* experiments with rats, measuring the blood volumetric flow rates is not feasible due to the small total volume of the rat’s blood. Therefore, creatinine removal rate $R_{cre}$[%] was used to investigate the performance of the membranes, as expressed in Eq. (8), where $C_{B(t)}$ is the creatinine concentration in the blood at time t.


$$R_{cre}=\frac{C_{B(0)}-C_{B(t)}}{C_{B(0)}}\times 100[\;\%]$$ (8)

*Ex vivo* experiments with SD rats were conducted using three-layer devices to confirm that the *ex vivo* results are comparable to the *in vitro* experiments. The thickness of the PES membranes was set based on the findings from the *in vitro* experiments.

The experimental protocol was approved by the Laboratory Animal Care and Use Committee in accordance with Keio University guidelines (14036-(5)). The experiments were conducted by researchers who had completed animal care and use training and had received instruction in surgical procedures.

SD rats (CLEA Japan, Inc., Tokyo, Japan), aged 32–36 weeks, were used for the experiments. First, the rats were anesthetized with 1.8% isoflurane (Mylan Seiyaku Ltd., Tokyo, Japan) via inhalation anesthesia (400 Anaesthesia, Zejtun, Malta). The device connection areas around the lower abdomen and thighs were shaved. After confirming the anesthesia state thoroughly, the skin and fascia were incised using a surgical scalpel, and the surrounding tissues and fat around the femoral artery and vein were carefully dissected to expose the blood vessels.

Micro-Renathane polyurethane tubes with outer and inner diameters of 0.94 mm and 0.58 mm, respectively (MRE037, Braintree Scientific Inc., Braintree, MA, USA), were used to access the blood vessels. The tubes were connected to the device via a Surflo indwelling needle (20G) with outer and inner diameters of 1.1 mm and 0.8 mm, respectively (SR-OT2032CP, Terumo Corporation, Tokyo, Japan). The device and tubing were filled with a solution of saline and heparin (Mochida Pharmaceutical Co., Ltd., Tokyo, Japan) in a 9:1 ratio to prevent blood coagulation within the device.

Both the femoral artery and vein were ligated while clamped a few centimeters away from the ligation points. The vessels were incised with a scalpel between the ligated and clamped points, and the tubes were inserted into the vessels toward the clamped points. Before insertion, the edges of the tubes were cut at an angle to create pointed tips. The blood vessels and tubes were secured by ligating them from the outside of the vessels. After ensuring the fixation was secure, the clamps were released, allowing blood to circulate through the device. These procedures were completed within 1 hour of initiating anesthesia.

After the blood pressure stabilized, blood samples were taken every 15 min and the creatinine concentration was measured. The creatinine removal rate R_cre in eq. (8) were compared to those of in vitro experiments, where the initial creatinine concentration of the simulated blood was adjusted to match that of rat blood, and the pressure difference between P_Bin and P_Bout was set to 100 mmHg, which is comparable to rat blood pressure. The number of trials for both *ex vivo* and *in vivo* experiments was 3 each.

Throughout the experiments, the conditions of the rats were monitored. At the end of the experiment, the concentration of the anesthetic was increased to 4.0%. After confirming cardiac arrest, the femoral artery was severed, and euthanasia was performed by exsanguination. The maximum duration of the experiments was 4 h before euthanasia. In these experiments, all the three rats reached the designed endpoint.

## Results and discussion

### ESEM analysis

**Fig 5:**
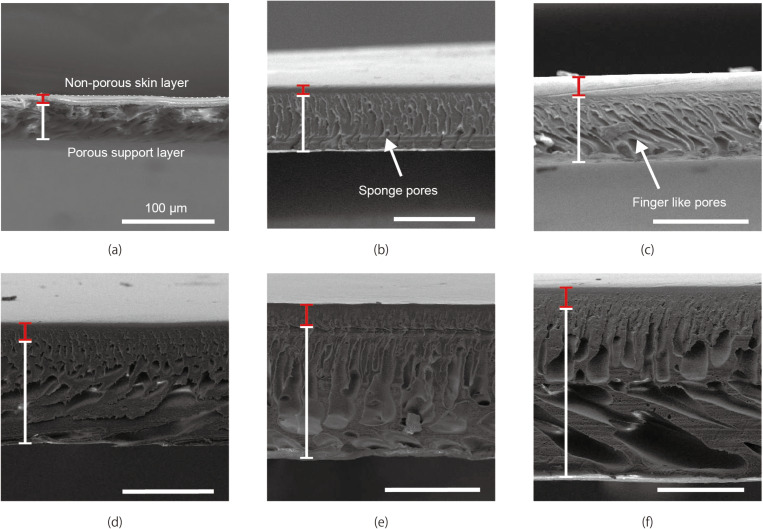
Cross sectional ESEM images of the PES membranes: (a) 50 μm, (b)100 μm (c)125 μm, (d)150 μm (e)200 μm, (f)250 μm. The scale bar represents 100μm.

**Fig 6:**
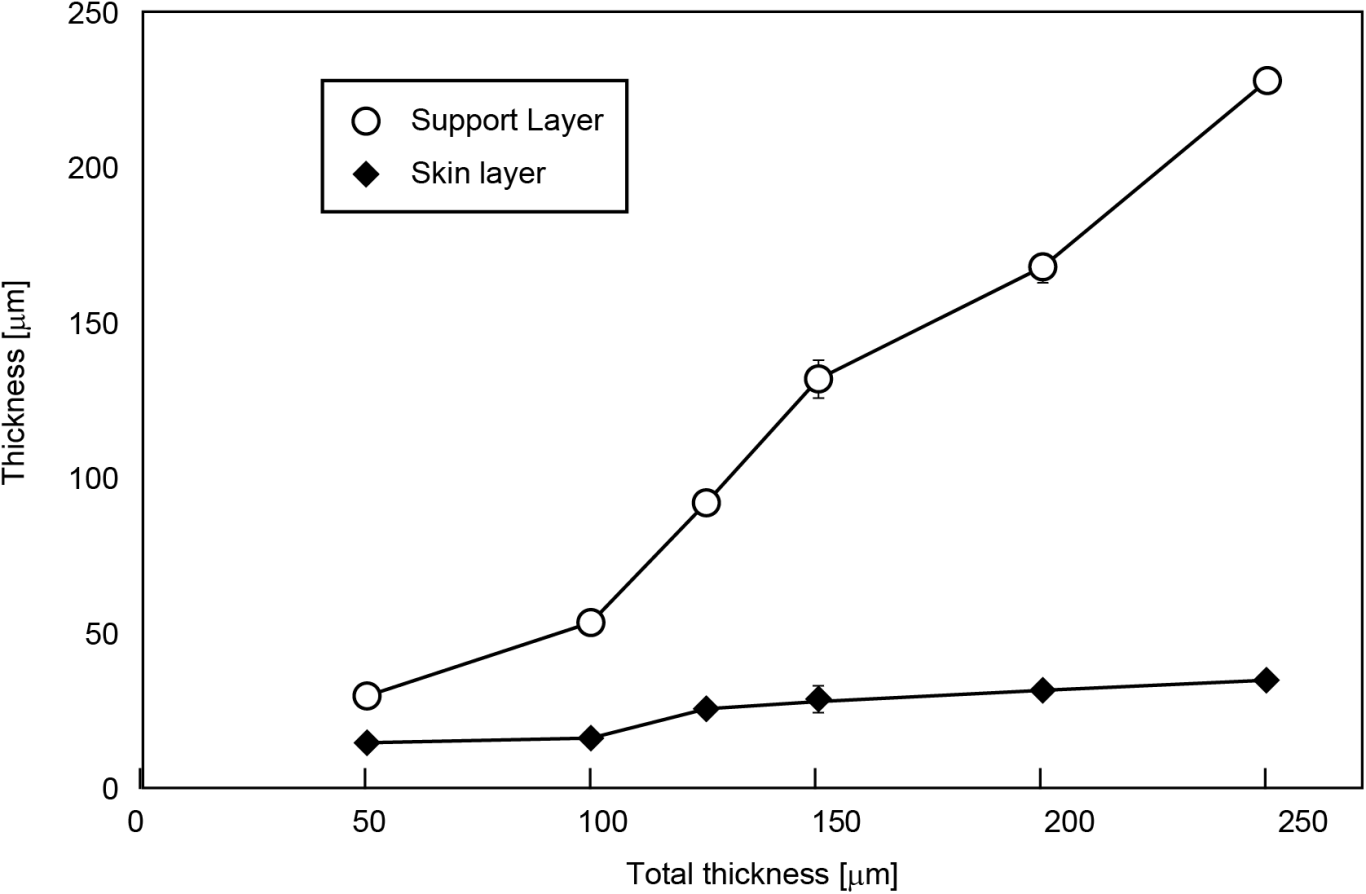
Thickness of skin layer and support layer at each film thickness.

Cross-sectional views of PES membranes with thicknesses from 50 μm to 250 μm are shown in Fig 5. The skin layer and the support layer can be easily recognized in the images. The skin layer thickness increased with the total thickness whereas the ratio of the skin layer to the support layer decreased, as shown in Fig 6. When the thickness of the PES membranes exceeded 125 μm, the support layer was found to have 2 layers with different microstructures; a layer with finger-like pores under a layer with spongy pores. (see Figs 5(c,5d,5e,5f).

### Pressure resistance tests

Three devices were tested for each thickness. The device with 40-µm-thick PES flat-sheet membrane ruptured at 41.5 ± 4.6 kPa (311 mmHg), while the membranes with a thickness larger than 80 µm successfully resisted 66.7 kPa (500 mmHg). One of the 80-µm-thick PES devices ruptured at 77 kPa (576 mmHg) and the other devices resisted 88 kPa (660 mmHg), which was the detection limit of the polygraph. The 120 and 160 μm flat-sheet PES membranes did not rupture at pressures at 88 kPa. Therefore, it was experimentally verified that the thickness of the PES flat-sheet membranes needs to be larger than 80 µm.

**Fig 7:**
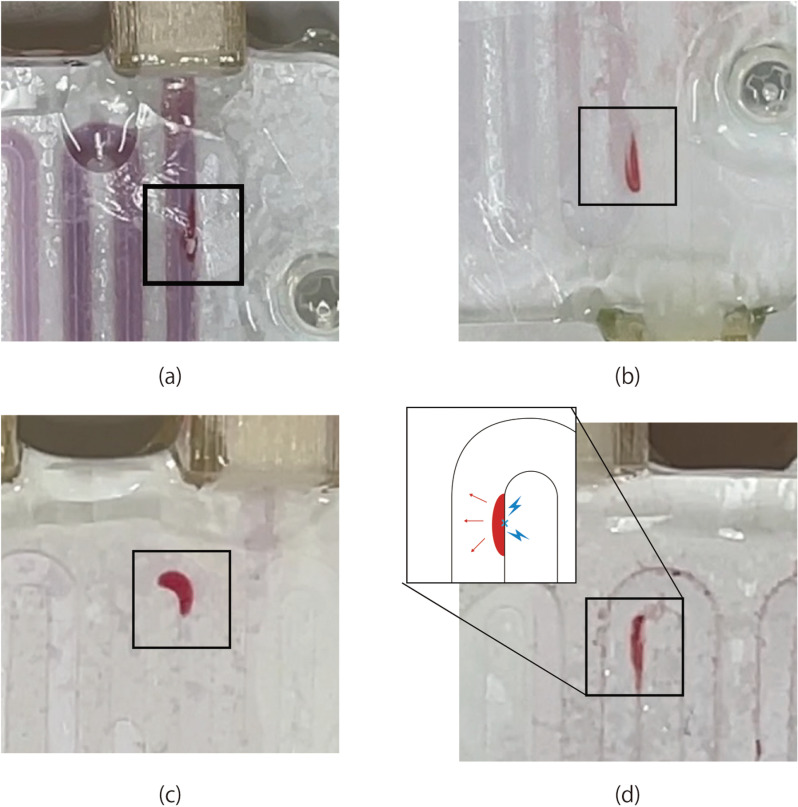
Photographs of PES flat-sheet membrane after the membrane rupture for the thickness of (a) 40, (b) 80, (c) 120, and (d) 160 μm.

Fig 7 shows the failure mode of the membranes. In case of the 120 and 160 μm flat-sheet PES membranes, the applied pressure increased until the membranes ruptured beyond the detection limit of the used polygraph. When the membranes rupture, blood (red in the photographs) leaks into the channels for filtrates. In all devices, the rupture was observed near the edge of the micro-channels, where the maximum stress is considered to be applied.

### Dialysis performance

**Fig 8:**
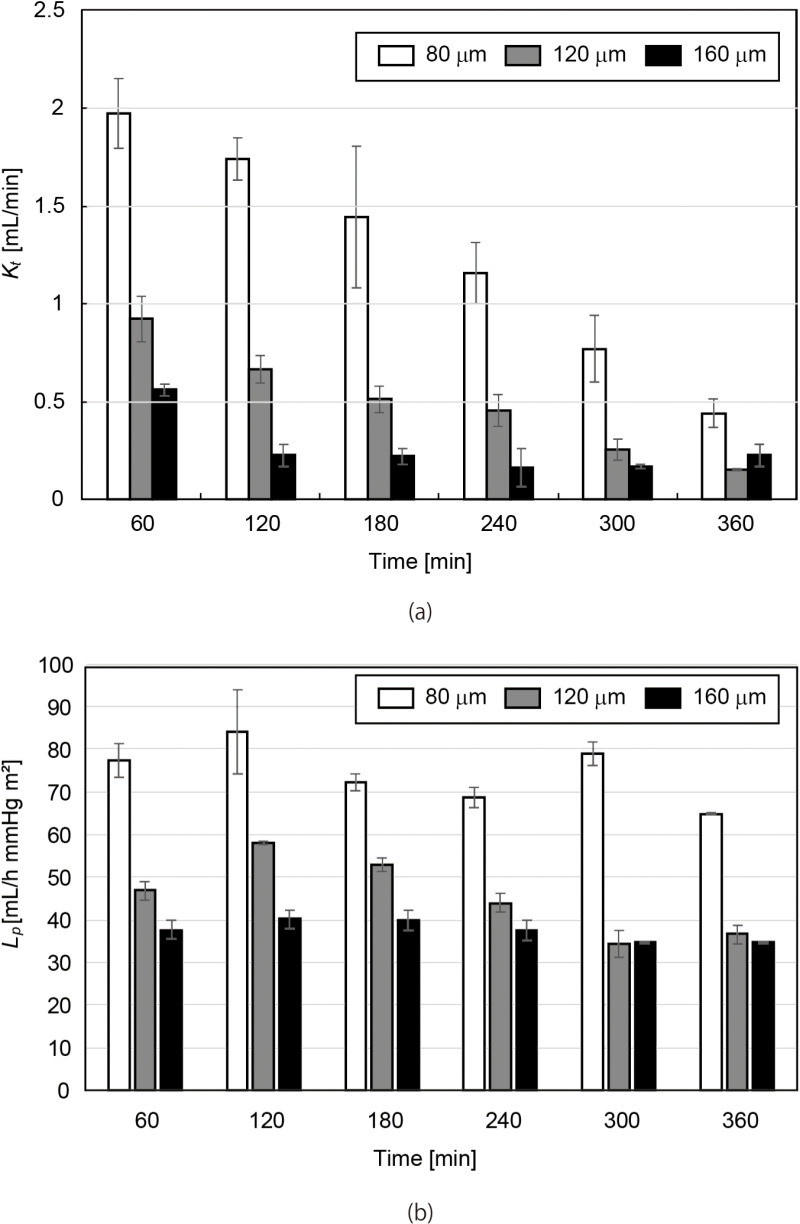
(a) The creatinine clearance and (b) the filtration coefficient as a function of the time. Error bar: means± S.D. n = 3. The commercially available dialyzers were reported to have $L_{p}$ of 20–60 mL/h mmHg m2 [25].

Fig 8(a),8(b) show the temporal variation of creatinine clearance $K_{t}$ and filtration coefficient $L_{p}$ with the membrane thickness of 80, 120, and 160 μm, respectively. $K_{t}$ decreased over time since the difference between the creatine concentration of the simulated blood and that of the dialysate decreased with time. $L_{p}$ decreases with the membrane thickness. The 80-μm-thick membrane showed good permeability, while the commercially available dialyzers were reported to have $L_{p}$ of 20–60 mL/h mmHg m^2^ [25].

### Creatinine removal rate for ex vivo experiments

**Fig 9:**
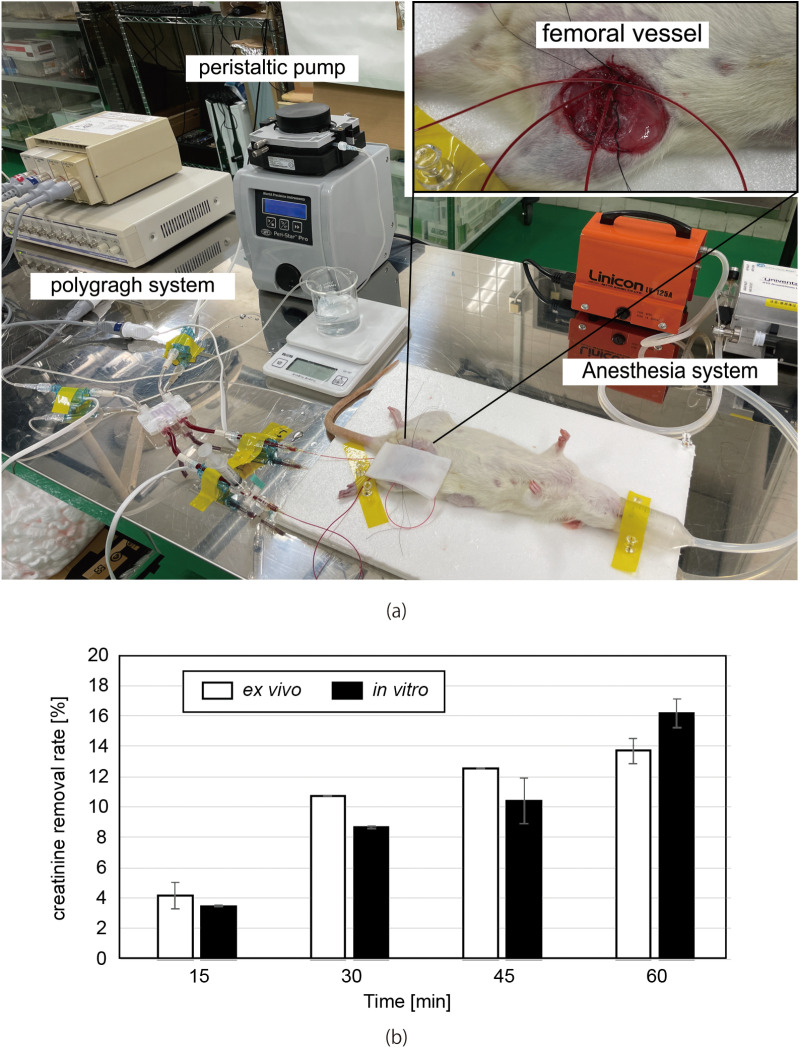
(a) Photograph of an *ex vivo* experiment with SD rats. The animal is under anesthesia. The device is connected to femoral artery and vein. $P_{Bin}$, $P_{Bout}$, $P_{Din}$ and $P_{Dout}$ are continuously measured with polygraph system. Blood is collected to measure the creatinine concentration. (b) Comparison of creatinine removal rate in *in vitro* and *ex vivo* experiments.

Based on the pressure resistance and dialysis tests, the target thickness of the PES membrane was set to 80 μm. The photo of the experiment is shown in Fig 9(a). As shown in Fig 9(b), the creatinine removal rate of *ex vivo* and *in vitro* experiments were found to be comparable. During the *ex vivo* experiments for 60 min, no severe effects by the rat blood were observed. We could not conduct experiments with rats under anesthesia for longer periods since the surgery takes time as well.

## Discussion

From the ESEM analyses, the thicknesses of both the skin layer and the support layer increase with the total membrane thickness. However, the increase in the skin layer is smaller than that of the support layer, indicating a trade-off between dialysis performance and mechanical strength.

Among the PES membranes with thicknesses of 40, 80, 120, and 160 μm tested for mechanical strength, the 40 μm membrane could not withstand 500 mmHg, the threshold set in this work. *In vitro* experiments with 80, 120, and 160-μm-thick PES membranes revealed that creatinine clearance $K_{t}$ and filtration coefficient $L_{p}$ decreased with the thickness. The filtration coefficient $L_{p}$ of commercially available dialyzers has been reported to range from 20 to 60 mL/h·mmHg·m² [25]. The PES membranes evaluated in this study exhibited comparable filtration properties, with the 80 μm membrane identified as the optimal choice, considering both mechanical strength and filtration efficiency.

In hemodialysis, membrane fouling due to the accumulation of organic materials from blood occurs over time, leading to a decline in dialysis performance [26]. This issue becomes particularly critical in implantable applications that require long-term operation, necessitating a thorough investigation of biofouling and blood coagulation. However, accurately replicating real-world conditions remains challenging due to the necessity of long-term animal studies.

Surface roughness has been reported as a key factor influencing biofouling [27–29]. For instance, an increase in the surface roughness of a polyacrylonitrile–polyamide 6/polyaniline-doped membrane was associated with enhanced hydrophilicity, leading to improved membrane performance in terms of flux and oil rejection [27]. Conversely, colloidal fouling on nanofiltration membranes has been shown to increase with nanoscale roughness [28]. Additionally, platelet adhesion and plasma protein adsorption on titanium alloy (Ti6Al4V) surfaces were found to increase with roughness, which must be minimized to prevent blood coagulation [29].

**Fig 10:**
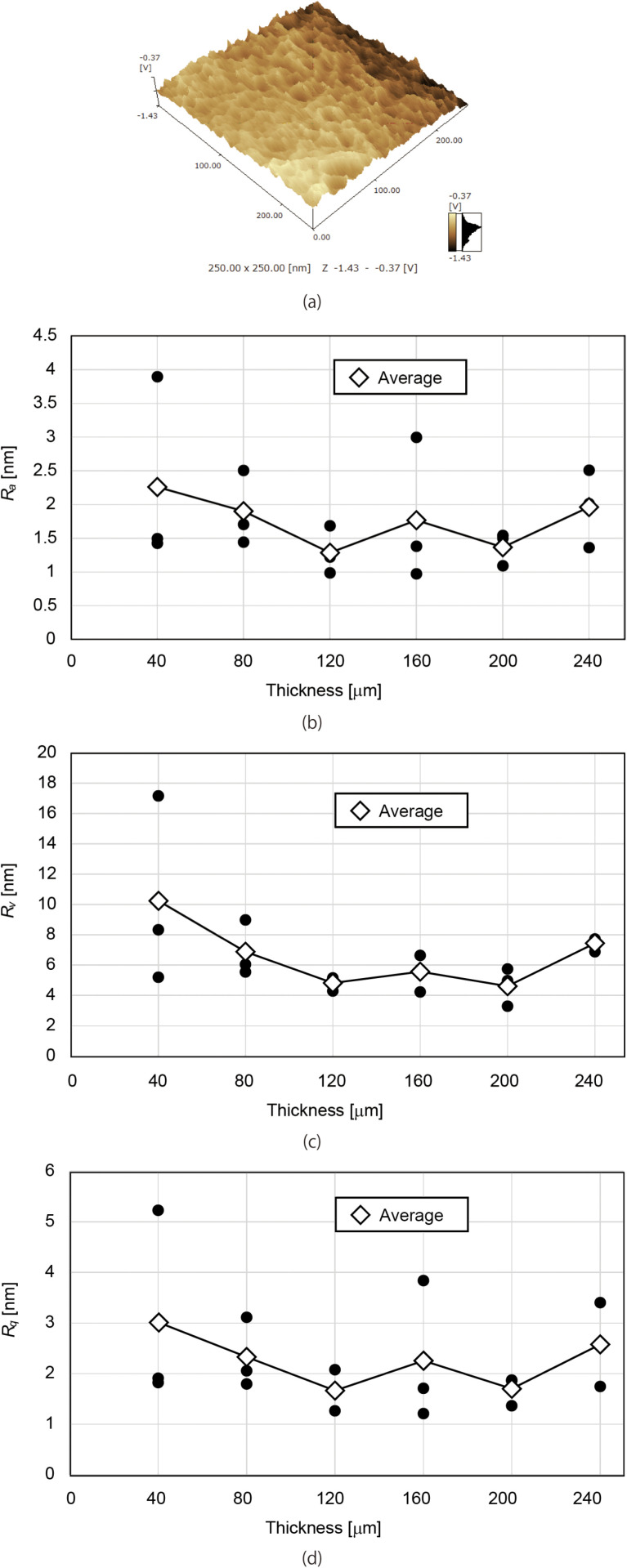
(a) AFM image of an 80-µm-thick membrane. (b) $R_{a}$, (c) $R_{v}$, and (d) $R_{q}$ values for membranes with thicknesses of 40, 80, 120, 160, 200, and 240 µm. Black dots indicate the measured values at three different locations, while the averaged values are represented by diamonds. No significant differences in surface roughness were observed with respect to membrane thickness. Furthermore, the measured roughness values were an order of magnitude lower than those reported in previous studies [27–29].

In this study, the surface roughness of PES membranes was analyzed using atomic force microscopy (AFM). The scanning area was set to 250 nm × 250 nm, and roughness measurements were conducted at three locations within a 100 nm × 100 nm region for each membrane. Fig 10(a) presents the AFM image of an 80-µm-thick membrane, while Figs 10(b–10d) depict the $R_{a}$, $R_{v}$, and $R_{q}$ values for membranes with thicknesses of 40, 80, 120, 160, 200, and 240 µm. The results indicate that all roughness parameters remained relatively constant across different thicknesses and were an order of magnitude lower than those reported in previous studies [27]. Therefore, membrane thickness is not considered a contributing factor to fouling.

In our 60-minute *ex vivo* experiments using Sprague-Dawley (SD) rats, no observable fouling was detected. However, longer-duration animal studies are required to further evaluate biofouling. For *in vivo* experiments, the selected animal model must be of sufficient size to accommodate the implantation of the device. Additionally, it is preferable to use an animal model for which experimental protocols, including surgery, anticoagulant drug administration, and monitoring, have been well established. Furthermore, to ensure the protection of the implanted device and the surrounding treated areas, the calm temperament of the animal is also an important consideration. Based on these criteria, we are currently designing *in vivo* studies using goats, following the protocol reported in previous research [30].

The filtration performance will be similar between humans and rats. However, due to differences in blood pressure, the amount of filtrate will vary. Additionally, hemodynamics related to coagulation differ between humans and animals. Nevertheless, conducting animal experiments is essential to validate the safety and effectiveness of the devices before proceeding with first-in-human trials.

## Conclusions

This study experimentally investigated the mechanical strength and dialysis efficiency of PES flat-sheet membranes utilized in our group’s multi-layered hemodiafiltration device with respect to thickness. The results of pressure resistance tests demonstrate that PES flat-sheet membranes with a thickness greater than 80 μm possess the mechanical strength to withstand pressures of 66.7 kPa (500 mmHg). The rupture of the membranes was observed at the edge of the channels, as expected. ESEM observation of PES flat-sheet membranes comprising two layers, a nanoporous skin layer and a porous support layer, revealed that the thickness of both layers increased with the total thickness, with the support layer increasing more rapidly. The PES membrane with a thickness of 80 µm was successfully used for *ex vivo* experiments. The appropriate thickness of the flat PES membrane for multi-layered hemodialysis and filtration devices was determined to be 80 µm.
