# Supplementary material for: Design of Polyether sulfone flat-sheet membranes for multi-layered hemodiafiltration devices
Source: PLoS One. 2025 Jun 20;20(6):e0324498. doi: 10.1371/journal.pone.0324498 (PMC12180728; doi:10.1371/journal.pone.0324498)
Supplement: Attachment — Submitted filename: Response to Reviewers.docx [file pone.0324498.s002.docx]

Dear reviewers,

We appreciate your time and effort to review our manuscript. We carefully considered your comments and revised our paper. The response and the corresponding revision are described below.

---

*Reviewer #1: The manuscript entitled "Design of Polyether Sulfone Flat-sheet Membranes for Multi-Layered Hemodiafiltration Devices" focuses on the design, development and utilization of poylethersulfone-based membranes and devices for hemodiafiltration applications. Generally, the article is well presented, however still some improvements are required to publish it in PLOS ONE. Following minor issues need to be addressed before acceptance.*

*1) What is novelty of this study?*

We appreciate your very important comment. In our previous manuscript, we failed to claim the novelty of this study. The novelty of this study is the design of flat PES membranes that achieve both sufficient mechanical strength and adequate filtration performance.

The ultimate goal of this study is to develop implantable hemofiltering devices. Most commercially available hemodialysis membranes are hollow fibers; however, the pressure loss across these fibers necessitates the use of an external pump to drive blood through the device. In implantable applications, where patients go about their daily lives outside of hospital beds, such pumps—which require electricity and add complexity and weight to the system—should be avoided. Additionally, hollow fibers may impose significant stress on the blood as it is distributed through tens of thousands of fibers before merging into a single flow. This process increases the likelihood of blood coagulation. Since implantable devices are intended for long-term use compared to conventional hemodialysis devices, minimizing blood coagulation is crucial. Therefore, we consider multilayered devices with flat filtering membranes to be a more suitable alternative for our application.

This study focuses on the filtering capacity and mechanical strength of the membranes. While blood coagulation and biofouling are critical concerns, investigating these aspects requires long-term in vivo experiments using large animals. We are currently developing experimental protocols for these in vivo studies and hope to report our findings in the near future.

We revised Abstract and Introduction as follows;

**Abstract**

Multilayered hemodialysis devices enable blood flow without the need for an external pump, whereas hollow fiber-type devices require a pump due to significant pressure loss across the fibers. This highlights a key advantage of multilayered devices for implantable applications, where a simpler and lighter system reduces the burden on patients. This study investigates the mechanical strength and dialysis efficiency of polyether sulfone (PES) flat-sheet membranes for multilayered devices. PES membranes, with thicknesses ranging from 40 to 160 µm, were prepared using the liquid inversion method, with thickness controlled via spin coating. The mechanical strength of the membranes was tested following the ISO 8637-1:2017 protocol, and membranes thicker than 80 µm were experimentally verified to withstand pressures of up to 500 mmHg, making them suitable for dialysis applications. Furthermore, the study demonstrates the successful use of 80 µm membranes in both in vitro and ex vivo experiments with rats, identifying this thickness as optimal for multilayered dialysis devices.

**INTRODUCTION**

----

Most of the commercially available dialyzers have hollow-fiber membranes due to the large surface-to-volume ratio, ease of manufacturing, and robustness against blood leak. The dialysis console pressurizes the blood with a pump up to 150 mmHg (20.0 kPa) and let the blood flow through the fibers, which are typically 200 μm in diameter and 16 - 30 cm in length. The thickness of the typical hollow-fiber membrane ranges from 10 to 50 μm [2].

There is a strong demand for implantable and wearable dialysis devices, as they reduce the frequency of hospital visits and significantly improve patients' quality of life. Such applications require a minimally complex system to enhance robustness and reduce the burden on patients. Hollow fiber-type devices necessitate an external pump to introduce blood due to the significant pressure loss across their narrow fibers. Such pumps require electricity, add complexity and weight to the system, and should be avoided. Additionally, hollow fibers may impose high mechanical stress on the blood as it passes through tens of thousands of fibers before merging into a single flow, increasing the likelihood of blood coagulation. Since implantable devices are intended for long-term use compared to conventional hemodialysis devices, minimizing blood coagulation is crucial for their effectiveness and safety.

Multi-layered hemodialysis devices, which are composed of micro-channels for blood and filtrates or dialysis fluids and flat-sheet dialysis membranes, can operate at low pressure due to the small pressure loss. Our group has been studying the multi-layered hemodialysis/filtration devices that can work under blood pressure without any external pumps for the implantable applications in mind [10-13]. Polyether sulfone (PES) is used for the membranes considering its high biocompatibility and low adhesiveness to biomaterials. The amount of diafiltration can be adjusted by the number of the layers.

*2) Line 27: What is difference between in vitro and ex vivo experiments?*

In the in vitro experiments, a saline solution containing albumin and creatinine, simulating blood, was used for filtration, while rat blood was used in the *ex vivo* experiments. In the in vitro experiments, the concentrations of albumin and creatinine can be adjusted to evaluate the filtration capacity of the membranes. The *ex vivo* experiments serve to verify that the membranes function effectively with rat blood, as well as with the simulated blood.

Given that the *ex vivo* experiments involve living rats, it is crucial to gather as much information as possible from the in vitro experiments beforehand. The rationale for conducting both in vitro and ex vivo experiments is outlined as follows:

***in vitro* experiments to evaluate dialysis performance**

Before testing the device in animals, as much information as possible should be gathered through *in vitro* experiments. The dialysis performance of the device is evaluated using the circuit, which is shown in Fig 4,

We also revised the description in ABSTACT (see the response to (1)).

*3) Figure 3: How can membranes of different geometrical shapes be compared to assess their pressures withstanding performance? Cylindrical shaped membranes can more efficiently withstand comparatively higher pressures than their flat shaped counterparts due to support from all directions in case of cylinders.*

We appreciate your comments. You are correct that cylindrical-shaped membranes, commonly known as hollow fiber membranes, exhibit greater mechanical strength and can be manufactured with a smaller thickness compared to flat membranes, as discussed in the **Mechanical Analysis** section.

However, flat membranes offer several advantages over hollow fibers, including the elimination of the need for an external pump to introduce blood into the device, reduced blood turbulence at the inlet and outlet, and greater design and fabrication flexibility.　The absence of a pump enhances operational robustness and reduces the burden on patients who rely on these devices in daily life. Additionally, the reduction in turbulence is particularly important, as turbulence can trigger blood coagulation, which is undesirable for long-term use in implantable devices.

We have added an explanation in INTRODUCTION to clarify the rationale behind designing multi-layered flat membranes. See the response to (1).

*4) Whole manuscript: There are many language errors specially involving numerous unnecessarily hyphenated words. Please remove hyphens throughout manuscript.*

We omitted the unnecessary hyphens.

*5) Was there any chance of blood contamination on applying this device?*

When the device is assembled properly, blood leakage and contamination does not occur. For long-term animal experiments, sterilization of the devices is mandatory to prevent infections.

*6) Discussion section needs to be further elaborated with references if available.*

We appreciate your advice. We added the evaluation of the filtration capacity of the manufactured membranes in comparison with commercially available dialyzer membranes [25]. Next, we explored the challenges of membrane fouling, acknowledging that accurately simulating real-world conditions remains difficult due to the necessity of long-term animal experiments. To address this, we analyzed the surface roughness of the membranes, a key factor reported to influence biofouling [27,28,29].

Additionally, we provided insights into the long-term in vivo experiments currently underway.

**DISCUSSION**

From the ESEM analyses, the thicknesses of both the skin layer and the support layer increase with the total membrane thickness. However, the increase in the skin layer is smaller than that of the support layer, indicating a trade-off between dialysis performance and mechanical strength.

Among the PES membranes with thicknesses of 40, 80, 120, and 160 μm tested for mechanical strength, the 40 μm membrane could not withstand 500 mmHg, the threshold set in this work. *In vitro* experiments with 80, 120, and 160-μm-thick PES membranes revealed that creatinine clearance $K_{t}$ and filtration coefficient $L_{p}$ decreased with the thickness. The filtration coefficient $L_{p}$ of commercially available dialyzers has been reported to range from 20 to 60 mL/h·mmHg·m² [25]. The PES membranes evaluated in this study exhibited comparable filtration properties, with the 80 μm membrane identified as the optimal choice, considering both mechanical strength and filtration efficiency.

In hemodialysis, membrane fouling due to the accumulation of organic materials from blood occurs over time, leading to a decline in dialysis performance [26]. This issue becomes particularly critical in implantable applications that require long-term operation, necessitating a thorough investigation of biofouling and blood coagulation. However, accurately replicating real-world conditions remains challenging due to the necessity of long-term animal studies.

Surface roughness has been reported as a key factor influencing biofouling [27–29]. For instance, an increase in the surface roughness of a polyacrylonitrile–polyamide 6/polyaniline-doped membrane was associated with enhanced hydrophilicity, leading to improved membrane performance in terms of flux and oil rejection [27]. Conversely, colloidal fouling on nanofiltration membranes has been shown to increase with nanoscale roughness [28]. Additionally, platelet adhesion and plasma protein adsorption on titanium alloy (Ti6Al4V) surfaces were found to increase with roughness, which must be minimized to prevent blood coagulation [29].

In this study, the surface roughness of PES membranes was analyzed using atomic force microscopy (AFM). The scanning area was set to 250 nm × 250 nm, and roughness measurements were conducted at three locations within a 100 nm × 100 nm region for each membrane. Figure 10 (a) presents the AFM image of an 80-µm-thick membrane, while Figures 10 (b–d) depict the $R_{a}$, $R_{v}$, and $R_{q}$ values for membranes with thicknesses of 40, 80, 120, 160, 200, and 240 µm. The results indicate that all roughness parameters remained relatively constant across different thicknesses and were an order of magnitude lower than those reported in previous studies [27]. Therefore, membrane thickness is not considered a contributing factor to fouling.

In our 60-minute *ex vivo* experiments using Sprague-Dawley (SD) rats, no observable fouling was detected. However, longer-duration animal studies are required to further evaluate biofouling. For *in vivo* experiments, the selected animal model must be of sufficient size to accommodate the implantation of the device. Additionally, it is preferable to use an animal model for which experimental protocols, including surgery, anticoagulant drug administration, and monitoring, have been well established. Furthermore, to ensure the protection of the implanted device and the surrounding treated areas, the calm temperament of the animal is also an important consideration. Based on these criteria, we are currently designing *in vivo* studies using goats, following the protocol reported in previous research [30].

The filtration performance will be similar between humans and rats. However, due to differences in blood pressure, the amount of filtrate will vary. Additionally, hemodynamics related to coagulation differ between humans and animals. Nevertheless, conducting animal experiments is essential to validate the safety and effectiveness of the devices before proceeding with first-in-human trials.

**References**

27. Faraji M, Nabavi SR, Salimi-Kenarib H. Fabrication of a PAN-PA6/PANI membrane using dual spinneret electrospinning followed by in situ polymerization for separation of oil-in-water emulsions. New J Chem. 2020, 44: 13488. Doi: 10.1039/D0NJ03231J
28. Linneweber J, Dohmen PM, Kerzscher U, Affeld K, Nosé Y, and Konertz W. The Effect of Surface Roughness on Activation of the Coagulation System and Platelet Adhesion in Rotary Blood Pumps. Artif Organs. 2007, 31 (5): 345–351. doi: 10.1111/j.1525-1594.2007.00391.x.
29. Vrijenhoek EM, Hong S., Elimelech M. Influence of membrane surface properties on initial rate of colloidal fouling of reverse osmosis and nanofiltration membranes. J Membr Sci. 2001, 188(1): 115-128. Doi:10.1016/S0376-7388(01)00376-3
30. Iizuka K, Katagiri N, Takewa Y, Tsukiya T, Mizuno T, Itamochi Y, Kumano K, Tatsumi E. Evaluation of the Novel Centrifugal Pump, CAPIOX SL, in Chronic Large Animal Experiments. Artif Organs. 2018 Aug;42(8):835-841. doi: 10.1111/aor.13100.

(a)

(b)

(c)

(d)

Fig 10. (a) AFM image of an 80-µm-thick membrane. (b) $R_{a}$, (c) $R_{v}$, and (d) $R_{q}$ values for membranes with thicknesses of 40, 80, 120, 160, 200, and 240 µm. Black dots indicate the measured values at three different locations, while the averaged values are represented by diamonds. No significant differences in surface roughness were observed with respect to membrane thickness. Furthermore, the measured roughness values were an order of magnitude lower than those reported in previous studies [27–29].

*7) Performance results on humans may be different due to presence of many components in blood stream. What results are expected for humans’ blood?*

The PES membranes allow only molecules and ions smaller than albumin to pass through. We consider that the filtration performance will be similar between humans and rats. However, due to differences in blood pressure, the amount of filtrate will vary. Additionally, hemodynamics related to coagulation differ between humans and animals. Nevertheless, conducting animal experiments is essential to validate the safety and effectiveness of the devices before proceeding with first-in-human trials. We added this in Discussion section (see the response to (6)).

*8) Prolonged filtration tests can help to assess fouling tendency of membranes.*

Thank you for your comments. Yes, investigating biofouling and blood coagulation is crucial to ensuring the long-term usability of the device. In *in vitro* experiments, it is impossible to fully replicate physiological blood conditions, as commercially available blood samples contain high concentrations of anticoagulants, making it difficult to accurately assess fouling.

To thoroughly examine these issues, long-term in vivo experiments using large animals such as goats and swine are necessary. Implanting the devices in these animals provides the necessary physiological environment for proper evaluation and ensures adequate protection for long-term use.

Currently, we are conducting in vivo experiments with the assistance of veterinarians; however, we are encountering various challenges, including surgical procedures and coagulation control through drug administration. We hope to report the findings of these long-term experiments in the near future.

In this paper, we investigated the surface roughness of the membranes, as it has been reported to influence biofouling properties. We have incorporated an explanation in the Discussion section (see the response to (6)).

*Reviewer #2: Comments on the manuscript entitled: Design of Polyether Sulfone Flat-sheet Membranes for Multi-Layered Hemodiafiltration Devices, PONE-D-24-51471
The following questions can be considered:
1. The abstract should contain answers to the following questions: What problem did you study and why is it important? What methods did you use? What were your main results? And what conclusions can you draw from your results? Please include in your abstract more specific and quantitative results when it suits broader audiences.*

We appreciate your comment. We revised the abstract following your precious advice as;

**Abstract**

Multilayered hemodialysis devices enable blood flow without the need for an external pump, whereas hollow fiber-type devices require a pump due to significant pressure loss across the fibers. This highlights a key advantage of multilayered devices for implantable applications, where a simpler and lighter system reduces the burden on patients. This study investigates the mechanical strength and dialysis efficiency of polyether sulfone (PES) flat-sheet membranes for multilayered devices. PES membranes, with thicknesses ranging from 40 to 160 µm, were prepared using the liquid inversion method, with thickness controlled via spin coating. The mechanical strength of the membranes was tested following the ISO 8637-1:2017 protocol, and membranes thicker than 80 µm were experimentally verified to withstand pressures of up to 500 mmHg, making them suitable for dialysis applications. Furthermore, the study demonstrates the successful use of 80 µm membranes in both in vitro and ex vivo experiments with rats, identifying this thickness as optimal for multilayered dialysis devices.

*2. To make the reader know you work clearly and quickly, the main objects of this study at the last paragraph in "Introduction" was suggested to be stated point by point.*

We agree with the reviewer. We modified the last paragraph of INTRODUCTION as follows;

In this paper, first, we discuss the differences between the flat-sheet membranes and hollow-fiber membranes used in the dialysis/filtration applications with respect to the mechanical properties. Then, we experimentally investigate the PES membranes with different thickness through pressure tests and *in vitro* and *ex vivo* hemodiafiltration experiments. The optimal membrane thickness will be deduced to design the implantable hemodiafiltration devices.

*3. The section on membrane preparation is vaguely written. Were the membranes prepared by the phase inversion method? In this case, after preparing the wet film, the wet film should be immediately immersed in deionized water. Whereas in the experimental section it is stated that the wet film was immersed in water for 24 hours. It is necessary to rewrite this section and write the details step by step.*

We appreciate your comment. We explained the manufacturing process in more details in Material and Device Fabrication as follows;

**Material and Device Fabrication**

The fabrication processes are shown in Fig 2. PES membranes are formed by the liquid inversion method [14]. First, PES with a molecular weight of 4800 (Sumitomo Chemical Co., Tokyo, Japan), polyethylene glycol with a molecular weight of 400 (Fuji-film Wako Chemical Corporation, Osaka, Japan), and dimethylacetamide (Fujifilm Wako Chemical Corporation, Osaka, Japan) were mixed in proportions of 17.5wt%, 14.6wt% and 67.9wt%, respectively, at 60 °C. The solution was then cooled to room temperature and stored. The membranes formed with PEG were experimentally verified to possess sufficient water permeability, which is essential for hemofiltration applications [12]. 250 mL of the PES casting solution was sufficient for forming 10 membranes.

Next, the PES casting solution was spin-coated onto a square glass substrate (100 mm × 100 mm), with the spinning speed determining the resulting membrane thickness. The volume of the solution was not precisely controlled during the spin-coating process. In our experiment, membrane thicknesses ranging from 40 to 250 µm were tested, corresponding to spinning speeds from 500 to 200 rpm. The spinning speed was maintained for 30 s. Within 5 s after the spin-coating process was completed, the substrate was immersed in deionized (DI) water for 24 h, allowing the PES membranes to form. The thickness of the membrane was measured using a digital micrometer.

For ESEM measurements, PES membranes with thicknesses of 50, 100, 150, 200, and 250 µm were evaluated, while 40, 80, 120, and 160 µm thick membranes were examined in the pressure resistance tests.

*4. What was the area of the membranes made by spin coating?*

The membrane was spin-coated onto a square glass plate (100 mm × 100 mm). We have added this information in Material and Device Fabrication (see the response to (3)).

*5. The volume of solution used to prepare the membranes should be stated.*

We prepared and stored 250 mL of the solution. The volume of the PES casting solution was not precisely controlled during the spin-coating process. 250 mL of the solution was sufficient for 10 membranes (see the response to (3)).

*6. Why wasn't PVP used instead of Polyethene glycol?*

In this study, the PES membranes are used for hemofiltration and must possess sufficient water permeability. The membranes formed with PEG were experimentally verified to meet these requirements [12]. In our previous work, PVP was used to form PES membranes [10]; however, the resulting membranes did not exhibit adequate water permeability. We have added this explanation in Material and Device Fabrication. (see the response to (3)).

10. Gu Y, Miki N. A microfilter utilizing a polyethersulfone porous membrane with nanopores. J Micromech Microeng. 2007, 17: 2308–2315. doi:10.1088/0960-1317/17/11/018.
12. To N, Sanada I, Ito H, Prihandana GS, Morita S, Kanno Y, et al. Water-permeable dialysis membranes for multi-layered microdialysis system. Front Bioeng Biotechnol. 2015, 3, 70. doi: 10.3389/fbioe.2015.00070.

*7. What was the thickness of fabricated membranes? How the authors measured this value? Details should be given in the experimental section.*

The spinning speed during the spin-coating process determined the resulting membrane thickness. In this study, the membrane thicknesses ranging from 40 to 250 µm were tested, corresponding to spinning speeds from 500 to 200 rpm. For ESEM measurements, PES membranes with thicknesses of 50, 100, 150, 200, and 250 µm were evaluated, while 40, 80, 120, and 160 µm thick membranes were examined in the pressure resistance tests.

The thickness of the membrane was measured using a digital micrometer. We have added this information in Material and Device Fabrication (see the response to (3)).

*8. For SEM images of the cross-section of membranes, the preparation method should be detailed. Were the membranes cut by a cutter?*

We appreciate your comment. We detailed the preparation process in ESEM Analysis;

**ESEM Analysis**

PES membranes consist of a skin layer with nanometer-sized pores and a support layer with micrometer-sized pores, which contribute to dialysis performance and mechanical strength, respectively. To investigate the relationship between the total membrane thickness and the thicknesses of the skin and support layers, we optically examined the PES membranes using an environmental scanning electron microscope (ESEM) (Inspect S50, FEI, USA). PES membranes with thicknesses of 50, 100, 150, 200, and 250 µm were analyzed.

For SEM imaging, the membranes were cut into 10 × 10 mm sections using a scalpel to expose the cross-section. The specimens were then attached to the stage with a conductive adhesive and dried for 1 hour. Subsequently, an osmium coating (5 nm thick) was applied before SEM measurement.

*9. During membrane testing, which side of the membrane was in contact with the dialysis solution: the support or the skin side?*

The skin side faces the blood layer and the support layer contact with the dialysis solution when used. We made this clear in Material and Device Fabrication.

Finally, the PES membrane, dialysate flow channel, and blood flow channel are assembled into a seven-layer device using cyanoacrylate adhesive (LOCTITE 4304/4305, Henkel Japan, Tokyo, Japan). The skin layer of the PES membrane faces the blood channel, while the support layer is in contact with the dialysate/filtrate channel. The assembled device is optically inspected to ensure that the adhesive is correctly applied.

*10. What was the method of measuring rejection? Details of the method with calibration curve must be given in the manuscript.*

We used saline solution mixed with albumin and creatinine and saline solution as the simulated blood and the dialysate, respectively. The simulated blood samples were collected from these circuits every hour for 6 hours and the concentrations of albumin and creatinine were measured using a dry clinical chemistry analyzer (Spot-Chem EZ SP-4430; Arkray, Kyoto, Japan). The analyzer's performance is reported to have a coefficient of variation (CV) of less than 4.5% for creatinine and less than 3.0% for albumin, as stated in the FDA 510(k) substantial equivalence determination (510(k) number: K053401). We consider that the measurement is accurate enough for our experiments. We added this information in *in vitro* experiments to evaluate dialysis performance.

The simulated blood samples were collected from these circuits every hour for 6 hours and the concentrations of albumin and creatinine were measured using a dry clinical chemistry analyzer (Spot-Chem EZ SP-4430; Arkray, Kyoto, Japan). The analyzer's performance is reported to have a coefficient of variation (CV) of less than 4.5% for creatinine and less than 3.0% for albumin, as stated in the FDA 510(k) substantial equivalence determination (510(k) number: K053401). The creatine clearance $K_{t}$ was calculated by Eq. (4) and the filtration coefficient $L_{p}$ was calculated by Eq. (5).

*11. In what time period was the amount of filtrate was measured to calculate the membrane flux? Details should be added in the experimental section.*

The experiments were conducted for six hours in the in vitro studies and four hours in the ex vivo studies. This information has been added to the experimental section. (see the response to (10)).

*12. The mechanical properties of the constructed membrane should be measured and the stress-strain curve should be given*

Thank you for your suggestions. In this study, we aimed to design membranes for our hemodialysis/filtration device, and followed the procedure outlined in *ISO 8637-1:2017, Extracorporeal Systems for Blood Purification – Part 1: Haemodialysers, Haemodiafilters, Haemofilters, and Haemoconcentrators* to discuss the strength of the membranes. We highlighted this point in the manuscript:

**Pressure Resistance Tests**

Pressure resistance tests were conducted to assess the mechanical strength of the PES flat-sheet membranes, following the procedure outlined in ISO 8637-1:2017, Extracorporeal Systems for Blood Purification – Part 1: Haemodialysers, Haemodiafilters, Haemofilters, and Haemoconcentrators.

*13. The roughness (Ra, Rv, Rq) of the membrane surface is key factor in fouling and can be calculated by AFM and added to the discussion section. The following paper is recommended: 10.1039/D0NJ03231J;*

Thank you for your comments and information of the article. We investigated the surface roughness of the fabricated PES membrane by AFM. The scanning areas were set to be 250 nm square and the measurement was performed at 3 locations in the area of 100 nm square on each sample with different thickness. As shown in Figure 10, the thickness did not affect the surface roughness. Surface roughness has been reported as a key factor influencing biofouling [27–29]. The measurement results indicate that all roughness parameters remained relatively constant across different thicknesses and were an order of magnitude lower than those reported in previous studies [27]. Therefore, membrane thickness is not considered a contributing factor to fouling.

**DISCUSSION**

From the ESEM analyses, the thicknesses of both the skin layer and the support layer increase with the total membrane thickness. However, the increase in the skin layer is smaller than that of the support layer, indicating a trade-off between dialysis performance and mechanical strength.

Among the PES membranes with thicknesses of 40, 80, 120, and 160 μm tested for mechanical strength, the 40 μm membrane could not withstand 500 mmHg, the threshold set in this work. *In vitro* experiments with 80, 120, and 160-μm-thick PES membranes revealed that creatinine clearance $K_{t}$ and filtration coefficient $L_{p}$ decreased with the thickness. The filtration coefficient $L_{p}$ of commercially available dialyzers has been reported to range from 20 to 60 mL/h·mmHg·m² [25]. The PES membranes evaluated in this study exhibited comparable filtration properties, with the 80 μm membrane identified as the optimal choice, considering both mechanical strength and filtration efficiency.

In hemodialysis, membrane fouling due to the accumulation of organic materials from blood occurs over time, leading to a decline in dialysis performance [26]. This issue becomes particularly critical in implantable applications that require long-term operation, necessitating a thorough investigation of biofouling and blood coagulation. However, accurately replicating real-world conditions remains challenging due to the necessity of long-term animal studies.

Surface roughness has been reported as a key factor influencing biofouling [27–29]. For instance, an increase in the surface roughness of a polyacrylonitrile–polyamide 6/polyaniline-doped membrane was associated with enhanced hydrophilicity, leading to improved membrane performance in terms of flux and oil rejection [27]. Conversely, colloidal fouling on nanofiltration membranes has been shown to increase with nanoscale roughness [28]. Additionally, platelet adhesion and plasma protein adsorption on titanium alloy (Ti6Al4V) surfaces were found to increase with roughness, which must be minimized to prevent blood coagulation [29].

In this study, the surface roughness of PES membranes was analyzed using atomic force microscopy (AFM). The scanning area was set to 250 nm × 250 nm, and roughness measurements were conducted at three locations within a 100 nm × 100 nm region for each membrane. Figure 10 (a) presents the AFM image of an 80-µm-thick membrane, while Figures 10 (b–d) depict the $R_{a}$, $R_{v}$, and $R_{q}$ values for membranes with thicknesses of 40, 80, 120, 160, 200, and 240 µm. The results indicate that all roughness parameters remained relatively constant across different thicknesses and were an order of magnitude lower than those reported in previous studies [27]. Therefore, membrane thickness is not considered a contributing factor to fouling.

In our 60-minute *ex vivo* experiments using Sprague-Dawley (SD) rats, no observable fouling was detected. However, longer-duration animal studies are required to further evaluate biofouling. For *in vivo* experiments, the selected animal model must be of sufficient size to accommodate the implantation of the device. Additionally, it is preferable to use an animal model for which experimental protocols, including surgery, anticoagulant drug administration, and monitoring, have been well established. Furthermore, to ensure the protection of the implanted device and the surrounding treated areas, the calm temperament of the animal is also an important consideration. Based on these criteria, we are currently designing *in vivo* studies using goats, following the protocol reported in previous research [30].

The filtration performance will be similar between humans and rats. However, due to differences in blood pressure, the amount of filtrate will vary. Additionally, hemodynamics related to coagulation differ between humans and animals. Nevertheless, conducting animal experiments is essential to validate the safety and effectiveness of the devices before proceeding with first-in-human trials.

**References**

27. Faraji M, Nabavi SR, Salimi-Kenarib H. Fabrication of a PAN-PA6/PANI membrane using dual spinneret electrospinning followed by in situ polymerization for separation of oil-in-water emulsions. New J Chem. 2020, 44: 13488. Doi: 10.1039/D0NJ03231J
28. Linneweber J, Dohmen PM, Kerzscher U, Affeld K, Nosé Y, and Konertz W. The Effect of Surface Roughness on Activation of the Coagulation System and Platelet Adhesion in Rotary Blood Pumps. Artif Organs. 2007, 31 (5): 345–351. doi: 10.1111/j.1525-1594.2007.00391.x.
29. Vrijenhoek EM, Hong S., Elimelech M. Influence of membrane surface properties on initial rate of colloidal fouling of reverse osmosis and nanofiltration membranes. J Membr Sci. 2001, 188(1): 115-128. Doi:10.1016/S0376-7388(01)00376-3
30. Iizuka K, Katagiri N, Takewa Y, Tsukiya T, Mizuno T, Itamochi Y, Kumano K, Tatsumi E. Evaluation of the Novel Centrifugal Pump, CAPIOX SL, in Chronic Large Animal Experiments. Artif Organs. 2018 Aug;42(8):835-841. doi: 10.1111/aor.13100.

(a)

(b)

(c)

(d)

Fig 10. (a) AFM image of an 80-µm-thick membrane. (b) $R_{a}$, (c) $R_{v}$, and (d) $R_{q}$ values for membranes with thicknesses of 40, 80, 120, 160, 200, and 240 µm. Black dots indicate the measured values at three different locations, while the averaged values are represented by diamonds. No significant differences in surface roughness were observed with respect to membrane thickness. Furthermore, the measured roughness values were an order of magnitude lower than those reported in previous studies [27–29].

*14. Fouling is the key factor for membrane characterization. Flux measurement with time is necessary and calculation of fouling factors should be done.*

Thank you for your comments. Yes, investigating biofouling and blood coagulation is crucial to ensuring the long-term usability of the device. In *in vitro* experiments, it is impossible to fully replicate physiological blood conditions, as commercially available blood samples contain high concentrations of anticoagulants, making it difficult to accurately assess fouling.

To thoroughly examine these issues, long-term in vivo experiments using large animals such as goats and swine are necessary. Implanting the devices in these animals provides the necessary physiological environment for proper evaluation and ensures adequate protection for long-term use.

Currently, we are conducting in vivo experiments with the assistance of veterinarians. We hope to report the findings of these long-term experiments in the near future.

We have included this discussion in the DISCUSSION section (see the response to (13)).

*15. Comparison of the performance of the fabricated membrane with similar one reported in the literature should be done.*

Thank you for your suggestions. According to the review paper [25], the ultrafiltration rate$L_{p}$ of the commercially available membranes range from 20 to 60. Although we stated this information in the caption of Figure 8, we added this explanation in Discussion explicitly (see the response to (13)).

(b)

Fig 8. (a) The creatinine clearance and (b) the filtration coefficient as a function of the time. Error bar: means± S.D. n=3. The commercially available dialyzers were reported to have $L_{p}$ of 20 to 60 mL/h mmHg m^2^ [25].

25. Said N, Lau WJ, Ho YC, Lim SK, Zainol Abidin MN, Ismail AF, A review of commercial developments and recent laboratory research of dialyzers and membranes for hemodialysis application. Membranes. 2021, 11: 767. doi: 10.3390/membranes11100767.
