# Supplementary material for: Design of Polyether sulfone flat-sheet membranes for multi-layered hemodiafiltration devices
Source: PLoS One. 2025 Jun 20;20(6):e0324498. doi: 10.1371/journal.pone.0324498 (PMC12180728; doi:10.1371/journal.pone.0324498)
Supplement: Peer review history

# Author comment: Author response to Decision Letter 0

**DOI:** 10.1371/journal.pone.0324498.r001

25 Nov 2024

# Peer review: Decision Letter 0

**DOI:** 10.1371/journal.pone.0324498.r002

**Authors:** Peter Corridon

Dear Dr. Miki,

Thank you for submitting your manuscript to PLOS ONE. After careful consideration, we feel that it has merit but does not fully meet PLOS ONE’s publication criteria as it currently stands. Therefore, we invite you to submit a revised version of the manuscript that addresses the points raised during the review process.

Please submit your revised manuscript by Mar 10 2025 11:59PM. If you will need more time than this to complete your revisions, please reply to this message or contact the journal office at plosone@plos.org . When you're ready to submit your revision, log on to https://www.editorialmanager.com/pone/ and select the 'Submissions Needing Revision' folder to locate your manuscript file.

- A rebuttal letter that responds to each point raised by the academic editor and reviewer(s). You should upload this letter as a separate file labeled 'Response to Reviewers'.
- A marked-up copy of your manuscript that highlights changes made to the original version. You should upload this as a separate file labeled 'Revised Manuscript with Track Changes'.
- An unmarked version of your revised paper without tracked changes. You should upload this as a separate file labeled 'Manuscript'.

If you would like to make changes to your financial disclosure, please include your updated statement in your cover letter. Guidelines for resubmitting your figure files are available below the reviewer comments at the end of this letter.

If applicable, we recommend that you deposit your laboratory protocols in protocols.io to enhance the reproducibility of your results. Protocols.io assigns your protocol its own identifier (DOI) so that it can be cited independently in the future. For instructions see: https://journals.plos.org/plosone/s/submission-guidelines#loc-laboratory-protocols . Additionally, PLOS ONE offers an option for publishing peer-reviewed Lab Protocol articles, which describe protocols hosted on protocols.io. Read more information on sharing protocols at https://plos.org/protocols?utm_medium=editorial-email&utm_source=authorletters&utm_campaign=protocols .

We look forward to receiving your revised manuscript.

Kind regards,

Peter R. Corridon

Academic Editor

PLOS ONE

Journal Requirements:

When submitting your revision, we need you to address these additional requirements.

1. Please ensure that your manuscript meets PLOS ONE's style requirements, including those for file naming. The PLOS ONE style templates can be found at

https://journals.plos.org/plosone/s/file?id=wjVg/PLOSOne_formatting_sample_main_body.pdf and

https://journals.plos.org/plosone/s/file?id=ba62/PLOSOne_formatting_sample_title_authors_affiliations.pdf

2.  Please include your full ethics statement in the ‘Methods’ section of your manuscript file. In your statement, please include the full name of the IRB or ethics committee who approved or waived your study, as well as whether or not you obtained informed written or verbal consent. If consent was waived for your study, please include this information in your statement as well.

3. Please update your submission to use the PLOS LaTeX template. The template and more information on our requirements for LaTeX submissions can be found at http://journals.plos.org/plosone/s/latex.

[Note: HTML markup is below. Please do not edit.]

Reviewers' comments:

Reviewer's Responses to Questions

**Comments to the Author**

1. Is the manuscript technically sound, and do the data support the conclusions?

Reviewer #1: Yes

Reviewer #2: Yes

**********

2. Has the statistical analysis been performed appropriately and rigorously?

Reviewer #1: Yes

Reviewer #2: Yes

**********

3. Have the authors made all data underlying the findings in their manuscript fully available?

The PLOS Data policy

Reviewer #1: Yes

Reviewer #2: No

**********

4. Is the manuscript presented in an intelligible fashion and written in standard English?

Reviewer #1: Yes

Reviewer #2: No

**********

Reviewer #1: The manuscript entitled "Design of Polyether Sulfone Flat-sheet Membranes for Multi-Layered Hemodiafiltration Devices" focuses on the design, development and utilization of poylethersulfone-based membranes and devices for hemodiafiltration applications. Generally, the article is well presented, however still some improvements are required to publish it in PLOS ONE. Following minor issues need to be addressed before acceptance.

1) What is novelty of this study?

2) Line 27: What is difference between in vitro and ex vivo experiments?

3) Figure 3: How can membranes of different geometrical shapes be compared to assess their pressures withstanding performance? Cylindrical shaped membranes can more efficiently withstand comparatively higher pressures than their flat shaped counterparts due to support from all directions in case of cylinders.

4) Whole manuscript: There are many language errors specially involving numerous unnecessarily hyphenated words. Please remove hyphens throughout manuscript.

5) Was there any chance of blood contamination on applying this device?

6) Discussion section needs to be further elaborated with references if available.

7) Performance results on humans may be different due to presence of many components in blood stream. What results are expected for humans’ blood?

8) Prolonged filtration tests can help to assess fouling tendency of membranes.

Reviewer #2: Comments on the manuscript entitled: Design of Polyether Sulfone Flat-sheet Membranes for Multi-Layered Hemodiafiltration Devices, PONE-D-24-51471

The following questions can be considered:

1. The abstract should contain answers to the following questions: What problem did you study and why is it important? What methods did you use? What were your main results? And what conclusions can you draw from your results? Please include in your abstract more specific and quantitative results when it suits broader audiences.

2. To make the reader know you work clearly and quickly, the main objects of this study at the last paragraph in "Introduction" was suggested to be stated point by point.

3. The section on membrane preparation is vaguely written. Were the membranes prepared by the phase inversion method? In this case, after preparing the wet film, the wet film should be immediately immersed in deionized water. Whereas in the experimental section it is stated that the wet film was immersed in water for 24 hours. It is necessary to rewrite this section and write the details step by step.

4. What was the area of the membranes made by spin coating?

5. The volume of solution used to prepare the membranes should be stated.

6. Why wasn't PVP used instead of Polyethene glycol?

7. What was the thickness of fabricated membranes? How the authors measured this value? Details should be given in the experimental section.

8. For SEM images of the cross-section of membranes, the preparation method should be detailed. Were the membranes cut by a cutter?

9. During membrane testing, which side of the membrane was in contact with the dialysis solution: the support or the skin side?

10. What was the method of measuring rejection? Details of the method with calibration curve must be given in the manuscript.

11. In what time period was the amount of filtrate was measured to calculate the membrane flux? Details should be added in the experimental section.

12. The mechanical properties of the constructed membrane should be measured and the stress-strain curve should be given

13. The roughness (Ra, Rv, Rq) of the membrane surface is key factor in fouling and can be calculated by AFM and added to the discussion section. The following paper is recommended: 10.1039/D0NJ03231J;

14. Fouling is the key factor for membrane characterization. Flux measurement with time in necessary and calculation of fouling factors should be done.

15. Comparison of the performance of the fabricated membrane with similar one reported in the literature should be done.

**********

what does this mean? ). If published, this will include your full peer review and any attached files.

If you choose “no”, your identity will remain anonymous but your review may still be made public.

**Do you want your identity to be public for this peer review?** For information about this choice, including consent withdrawal, please see our Privacy Policy

Reviewer #1: **Yes: ** Muhammad Sarfraz

Reviewer #2: No

**********

[NOTE: If reviewer comments were submitted as an attachment file, they will be attached to this email and accessible via the submission site. Please log into your account, locate the manuscript record, and check for the action link "View Attachments". If this link does not appear, there are no attachment files.]

While revising your submission, please upload your figure files to the Preflight Analysis and Conversion Engine (PACE) digital diagnostic tool, https://pacev2.apexcovantage.com/ . PACE helps ensure that figures meet PLOS requirements. To use PACE, you must first register as a user. Registration is free. Then, login and navigate to the UPLOAD tab, where you will find detailed instructions on how to use the tool. If you encounter any issues or have any questions when using PACE, please email PLOS at figures@plos.org . Please note that Supporting Information files do not need this step.

# Author comment: Author response to Decision Letter 1

**DOI:** 10.1371/journal.pone.0324498.r003

5 Mar 2025

We appreciate your time and effort to review our manuscript. We carefully considered your comments and revised our paper. The response and the corresponding revision are described in "Response to Reviewer" file.

Attachment Submitted filename: Response to Reviewers.docx

# Peer review: Decision Letter 1

**DOI:** 10.1371/journal.pone.0324498.r004

**Authors:** Muhammad Shakaib

Design of Polyether Sulfone Flat-sheet Membranes for Multi-Layered Hemodiafiltration Devices

PONE-D-24-51471R1

Dear Dr. Miki,

We’re pleased to inform you that your manuscript has been judged scientifically suitable for publication and will be formally accepted for publication once it meets all outstanding technical requirements.

Within one week, you’ll receive an e-mail detailing the required amendments. When these have been addressed, you’ll receive a formal acceptance letter and your manuscript will be scheduled for publication.

An invoice will be generated when your article is formally accepted. Please note, if your institution has a publishing partnership with PLOS and your article meets the relevant criteria, all or part of your publication costs will be covered. Please make sure your user information is up-to-date by logging into Editorial Manager at Editorial Manager®  and clicking the ‘Update My Information' link at the top of the page. If you have any questions relating to publication charges, please contact our Author Billing department directly at authorbilling@plos.org.

If your institution or institutions have a press office, please notify them about your upcoming paper to help maximize its impact. If they’ll be preparing press materials, please inform our press team as soon as possible -- no later than 48 hours after receiving the formal acceptance. Your manuscript will remain under strict press embargo until 2 pm Eastern Time on the date of publication. For more information, please contact onepress@plos.org.

Kind regards,

Muhammad Shakaib, PhD

Academic Editor

PLOS ONE

Additional Editor Comments (optional):

Reviewers' comments:

Reviewer's Responses to Questions

**Comments to the Author**

Reviewer #1: All comments have been addressed

Reviewer #2: All comments have been addressed

**********

2. Is the manuscript technically sound, and do the data support the conclusions?

Reviewer #1: Yes

Reviewer #2: Yes

**********

3. Has the statistical analysis been performed appropriately and rigorously?

Reviewer #1: N/A

Reviewer #2: Yes

**********

4. Have the authors made all data underlying the findings in their manuscript fully available?

The PLOS Data policy

Reviewer #1: Yes

Reviewer #2: Yes

**********

5. Is the manuscript presented in an intelligible fashion and written in standard English?

Reviewer #1: Yes

Reviewer #2: Yes

**********

Reviewer #1: Dear authors,

Thank you for addressing all comments. Revised article can now be accepted for publication in PLOS ONE.

Reviewer #2: The authors replied all comments properly and manuscript can be considered for publication in the PLOS One.

**********

what does this mean? ). If published, this will include your full peer review and any attached files.

If you choose “no”, your identity will remain anonymous but your review may still be made public.

**Do you want your identity to be public for this peer review?** For information about this choice, including consent withdrawal, please see our Privacy Policy

Reviewer #1: **Yes: ** Muhammad Sarfraz

Reviewer #2: No

**********

# Editor report: Acceptance letter

**DOI:** 10.1371/journal.pone.0324498.r005

**Authors:** Muhammad Shakaib

PONE-D-24-51471R1

PLOS ONE

Dear Dr. Miki,

I'm pleased to inform you that your manuscript has been deemed suitable for publication in PLOS ONE. Congratulations! Your manuscript is now being handed over to our production team.

At this stage, our production department will prepare your paper for publication. This includes ensuring the following:

* All references, tables, and figures are properly cited

* All relevant supporting information is included in the manuscript submission,

* There are no issues that prevent the paper from being properly typeset

You will receive further instructions from the production team, including instructions on how to review your proof when it is ready. Please keep in mind that we are working through a large volume of accepted articles, so please give us a few days to review your paper and let you know the next and final steps.

Lastly, if your institution or institutions have a press office, please let them know about your upcoming paper now to help maximize its impact. If they'll be preparing press materials, please inform our press team within the next 48 hours. Your manuscript will remain under strict press embargo until 2 pm Eastern Time on the date of publication. For more information, please contact onepress@plos.org.

If we can help with anything else, please email us at customercare@plos.org.

Thank you for submitting your work to PLOS ONE and supporting open access.

Kind regards,

PLOS ONE Editorial Office Staff

on behalf of

Dr. Muhammad Shakaib

Academic Editor

PLOS ONE
